# Photodynamic priming with triple-receptor targeted nanoconjugates that trigger T cell-mediated immune responses in a 3D *in vitro* heterocellular model of pancreatic cancer

**DOI:** 10.1515/nanoph-2021-0304

**Published:** 2021-08-18

**Authors:** Pushpamali De Silva, Shazia Bano, Brian W. Pogue, Kenneth K. Wang, Edward V. Maytin, Tayyaba Hasan

**Affiliations:** Wellman Center for Photomedicine, Massachusetts General Hospital, Harvard Medical School, Boston, MA, 02114, USA.; Wellman Center for Photomedicine, Massachusetts General Hospital, Harvard Medical School, Boston, MA, 02114, USA.; Thayer School of Engineering, Dartmouth College, Hanover, NH, 03755, USA; Division of Gastroenterology and Hepatology, Mayo Clinic, Rochester, MN, 55905, USA; Departments of Dermatology and Biomedical Engineering, Cleveland Clinic, Cleveland, OH, 44195, USA; Wellman Center for Photomedicine, Massachusetts General Hospital, Harvard Medical School, 40 Blossom Street, BAR 314A, Boston, MA, 02114, USA; Division of Health Sciences and Technology, Massachusetts Institute of Technology, Harvard University, Cambridge, MA, 02139, USA

**Keywords:** immunogenic cell death, multitargeting, photo-dynamic therapy, photoimmuno-nano-conjugates, T cell priming, tumor heterogeneity

## Abstract

Photodynamic priming (*PDP*), a collateral effect of photodynamic therapy, can transiently alter the tumor microenvironment (TME) beyond the cytotoxic zone. Studies have demonstrated that *PDP* increases tumor permeability and modulates immune-stimulatory effects by inducing immunogenic cell death, via the release of damage-associated molecular patterns and tumor-associated antigens. Pancreatic ductal adenocarcinoma (PDAC) is one of the deadliest of cancers with a stubborn immunosuppressive TME and a dense stroma, representing a challenge for current molecular targeted therapies often involving macromolecules. We, therefore, tested the hypothesis that PDP’s TME modulation will enable targeted therapy and result in immune stimulation. Using triple-receptor-targeted photoimmuno-nanoconjugate (TR-PINs)-mediated *PDP*, targeting epidermal growth factor receptor, transferrin receptor, and human epidermal growth factor receptor 2 we show light dose-dependent TR-PINs mediated cytotoxicity inhuman PDA Ccells (MIAPaCa-2),co-cultured with human pancreatic cancer-associated fibroblasts (PCAFs) in spheroids. Furthermore, TR-PINs induced the expression of heat shock proteins (Hsp60, Hsp70), Calreticulin, and high mobility group box 1 in a light dose and time-dependent manner.TR-PINs-mediated T cell activation was observed in co-cultures of immune cells with the MIA PaCa-2-PCAF spheroids. Both CD4^+^ T and CD8^+^ T cells showed light dose and time-dependant antitumor reactivity by upregulating degranulation marker CD107a and interferon-gamma post-PDP. Substantial tumor cell death in immune cell-spheroid co-cultures by day 3 shows the augmentation by antitumor T cell activation and their ability to recognize tumors for a light dose-dependent kill. These data confirm enhanced destruction of heterogeneous pancreatic spheroids mediated by *PDP*-induced phototoxicity, TME modulation and increased immunogenicity with targeted nanoconstructs.

## 1 Introduction

Photodynamic therapy (PDT), a Food and Drug Administration (FDA)-approved anticancer therapy is based on the activation of a photosensitizer (PS) with an appropriate wavelength of light, typically red light. Reactive molecular species (RMS) generated from the photodynamic activation process then provide the cell killing and other tumor modulation effects [1]. While classically, this is thought to lead to photoablation of the tumor cells and subsequent cell death either by direct cytotoxicity or damage to the tumor vasculature, there is also a host of sub-lethal cell signaling changes that alter the tumor milieu. Responses to PDT may be modulated to a large extent by varying the light dose, PS concentration, and drug light interval (DLI). Due to its minimally invasive properties as a therapeutic modality, PDT holds great promise to be used in alternative treatments or in combination with other conventional anticancer treatments such as surgery, chemotherapy, or radiotherapy [2]. It is indeed approved for several indications by the regulatory authorities worldwide. PDT-activated immune responses are both local and extended systemically far beyond the irradiated site [3, 4].

Preclinical and clinical studies have demonstrated that PDT can affect both the innate and adaptive arms of the immune system [3, 5–7]. These immune-stimulatory effects occur through PDT’s ability to induce immunogenic cell death (ICD), which increases the immunogenicity of the tumor microenvironment (TME) by the release of damage-associated molecular patterns (DAMPs) and tumor-associated antigens (TAAs) [8, 9]. The degree of ICD by PDT greatly depends on the release of RMS [10]. PDT can induce a large amount of RMS production inside the cancer cells, thereby causing oxidative stress-based cell death. PDT generates DAMPs such as calreticulin (CRT), heat shock proteins (Hsp60, Hsp70, and Hsp90), high mobility group box 1(HMGB1), and extracellular ATP [8, 11, 12]. DAMPs and cytokines (such as tumor necrosis factor (TNF)-*α*, interleukin (IL)-6, and IL-1*β*) released from PDT treated cells cause acute inflammation and enhance infiltration of innate and adaptive immune cells to the irradiated tumor site [8, 13–18]. PDT enhances antigen presentation by professional antigen-presenting cells (APC), such as dendritic cells (DCs), whereby TAAs are processed and presented to cells of the adaptive immune system; especially T cells [9, 17, 19]. During PDT-mediated release of DAMPs and subsequent inflammation, APCs mature and migrate to the draining lymph nodes. This transition of DCs involves their activation via the upregulation of major histocompatibility class I and II molecules (MHC-I and MHC-II) and the costimulatory molecules CD80 and CD86 on their cell surfaces. Once DCs are activated they are efficient in priming CD4^+^ T helper cells and CD8^+^ cytotoxic T lymphocytes (CTLs) by the presentation of TAAs and initiate an effective adaptive immune response. Antigen-experienced CTLs may migrate to the tumor site to attack the remaining and/or metastasized tumor cells [9]. Overall, PDT may turn “immune silent” tumors into “immune responsive” tumors by inducing ICD and enhanced immunogenicity following it.

Recent evidence shows that a collateral effect of a sublethal dose of PDT termed photodynamic priming (*PDP*) [20], confers increased immunogenicity [3, 21] by priming multiple compartments in the TME. *PDP*-associated immune-stimulatory effects have been shown to enhance the infiltration of neutrophils and activated CTLs in the TME [3, 18, 21]. Also, our work in pancreatic ductal adenocarcinoma (PDAC) murine models demonstrated that *PDP* can prime multiple tumor compartments to enable a more potent and sustained antitumor chemotherapeutic effect [20] or chemotherapy dose reduction for improved tolerability [22]. PDAC is one of the most lethal cancers with a low response to treatment of any kind including immune therapies and a five-year survival rate of around 10% [23]. PDAC pathophysiology is challenging for current therapies as immunosuppressive desmoplastic stroma limits responsiveness to treatments including macromolecular targeting and immunotherapy [24–26].

Photoimmunoconjugates that target cell membrane molecules overexpressed by cancer cells create a combined photodynamic and receptor antagonist therapeutic agent for tumor-targeted, activatable photoimmunotherapy (PIT) [27].NIR-PIT induces ICD and expression and translocation of DAMPs followed by maturation of DCs, thus eliciting a host immune response against the tumor [28]. Combined with the molecular targeting ability of the receptor targeted nanoconstructs, PIT may be a powerful strategy for inducing ICD in cancer therapy. However, despite ongoing human trials (NCT02422979; PIT using a conjugate of the silicon phthalocyanine PS derivative IRDye700DX with cetuximab [Cet] [29]), complete tumor eradication is hampered by intratumoral receptor heterogeneity, leading to the survival of residual resistant tumor cells. The inability to target multiple receptors simultaneously is a clinical obstacle for optimal treatment outcomes due to the heterogeneity of tumors with multiple survival pathways being operative.

**Figure 1:**
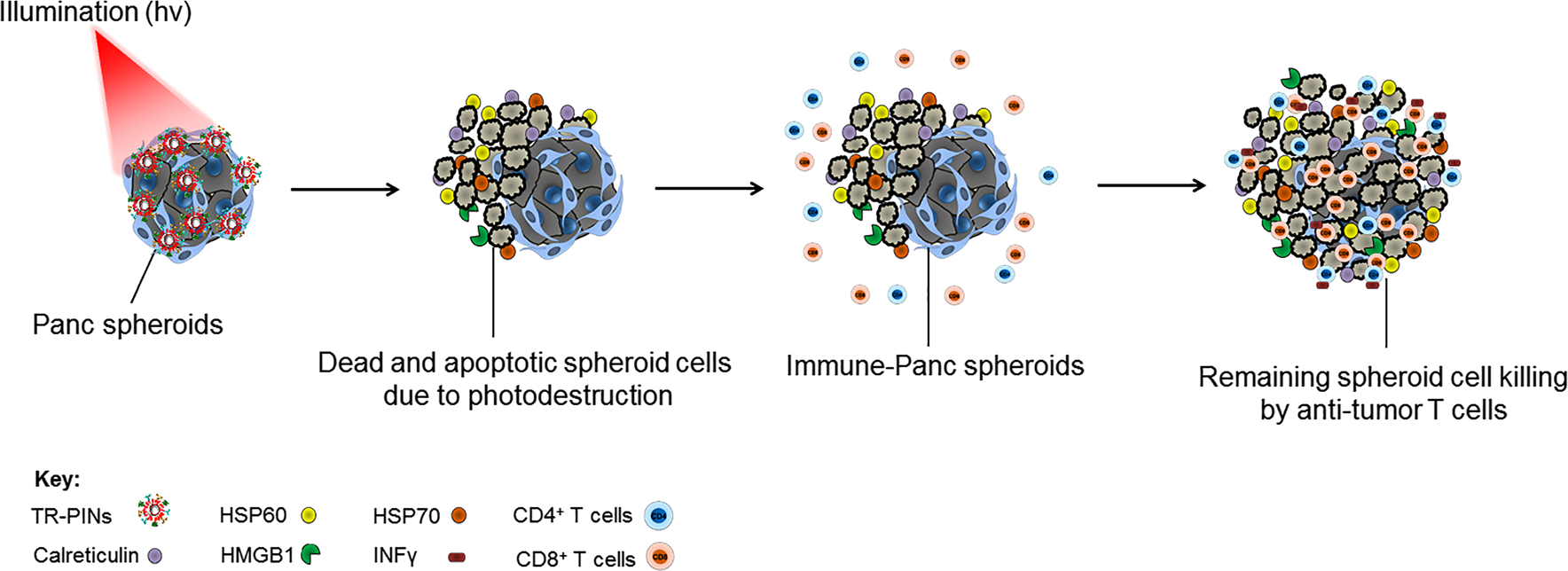
Schematic concept of the study. Photodynamic priming (PDP) induces the expression of HSP60, HSP70, Calreticulin, and HMGB1 in Panc spheroids, when treated with triple-receptor-targeted photoimmuno-nanoconjugates (TR-PINs). This highlights the direct tumor cell killing and the induction of immunogenic cell death by TR-PINs, enhancing the immunogenicity of spheroids. Upregulation of degranulation marker CD107a and interferon-gamma (IFN*γ*) in CD4^+^ T cells and CD8^+^ T cells demonstrates efficient T cell priming due to enhanced immunogenicity. The direct phototoxic effects of TR-PINs and photo-primed antitumor T cells show substantial tumor cell death, suggesting enhanced tumor killing.

Recently, we reported the targeting of multiple receptors on tumor cells to address heterogeneity-driven resistance to molecular targeted PDT. Red-activatable, triple-receptor-targeted photoimmuno-nanoconjugates (TR-PINs) platform conferred specificity for epidermal growth factor receptor (EGFR), transferrin receptor (TfR), and human epidermal growth factor receptor 2 (HER-2). Multi-targeting enhanced the specificity and overall completeness of PDT response in a heterogeneous tumor model of MIA PaCa-2 and T47D or SKOV-3 cells when compared to mono-targeting [30]. In this study, we set out to establish whether *PDP* multiple targeting of tumor cells can initiate an antitumor immune response by enhancing tumor immunogenicity (Figure 1) while taking care of the heterogeneous cell populations. Following covalent conjugation of functionalized ligands to the surface of photosensitizing liposomal nanoconstructs, the innovative binding of TR-PINs to the tumor cells was used to evaluate, PDT efficacy, associated immune-stimulatory effects, and the degree of ICD induction *in vitro* three-dimensional (3D) heterogeneous tumor model of PDAC and pancreatic cancer-associated fibroblasts (PCAFs). We quantified the expression of Hsp60, Hsp70, CRT, and HMGB1. We also investigated the efficient priming of T cells and their ability to perform further killing of malignant cells by co-culturing MIA PaCa-2 and PCAFs with allogenic human peripheral blood mononuclear cells (PBMC). For clarity, we term heterocellular MIA PaCa-2-PCAF spheroids as Pancreatic (Panc) spheroids and where Panc spheroids are cocultured with immune cells as Immune-Panc spheroids. TR-PINs were able to exert direct cytotoxic effects followed by enhanced ICD in Panc spheroids. We found marked enhancement of T cell priming and effective tumor cell killing in PDT treated Immune-Panc spheroids consistent with the triggering an effective immune response to TR-PINs mediated PDP.

## 2 Results and discussion

### 2.1 Design, preparation, and characterization of TR-PINs

**Figure 2:**
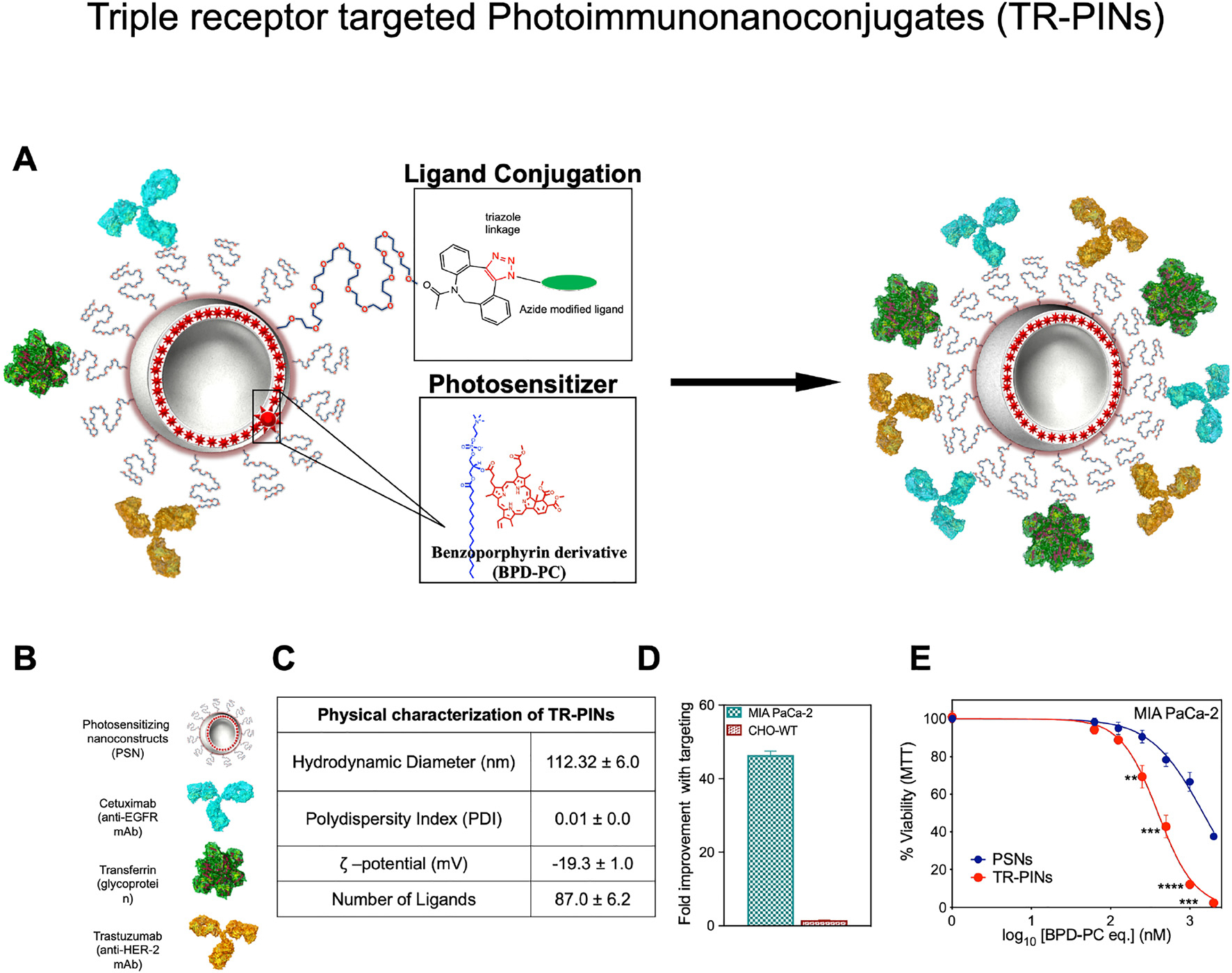
Schematic representation of the design of triple-receptor-targeted photoimmuno-nanoconjugates (TR-PINs). Design of triple-receptor-targeted photoimmuno-nanoconjugates (TR-PINs) (A) and the ligands (B) conjugated via a copper-free click chemistry approach. Physical characterization of the TR-PINs (C). TR-PINs exhibit a significant improvement in binding to MIA PaCa-2 cells (D). BPD-PC emission intensities measured via flow cytometry were used to analyze TR-PINs cellular binding as compared to the photosensitizing nanoconstructs (PSNs). Representative phototoxicity dose–response curves of the PSNs and the TR-PINs in MIA PaCa-2 monolayers (E). The NIR photodynamic activation regimen employed 690 nm light irradiation and 20 J/cm^2^ at 150 mW/cm^2^ (mean± SEM; *n* = 9–12 for a–c; one-way ANOVA with a Tukey post-test; ****P ≤ 0.0001, ***P ≤ 0.001, **P ≤ 0.01).

The preparation of TR-PINs (Figure 2(A)), is published [30]. Briefly, the liposomal photosensitizing nanoconstructs (PSNs) were formed, incorporating a lysophospholipid anchored variant of the hydrophobic photosensitizer benzoporphyrin derivative (BPD), within the liposomal bilayer. Lipidation of BPD had no impact on its absorption properties, as determined by the lack of any spectral shifts [31, 32]. The purified lipidated variant (BPD-PC) of PS was characterized by Matrix Assisted Laser Desorption/Ionization (MALDI) to verify molecular weight and by HPLC to assess purity [31, 32]. Moreover, BPD-PC containing photosensitizing liposomal nanoconstructs remained colloidally stable with the BPD inserted into the hydrophobic bilayer. When incubated with OVCAR-5 cells, these liposomal nanoconstructs demonstrated no PS leaching [32]. As such the lipid anchoring strategy adopted here, modulate the PS’s membrane stability, and promote nanoconstruct integrity. Three ligands, Cet, holo-transferrin (HT), and trastuzumab (TZ) (Figure 2(B)) were modified and conjugated to the surface of the PSNs [30, 32]. Figure 2(C) provides details of the physical characterizations that need to be carefully considered for the rational design of targeted nanoconstructs. TR-PINs exhibit an average hydrodynamic size of 112.32 ± 6.0, with the polydispersity indices 0.01 ± 0.02 suggesting a narrow size distribution of liposomal nanoconstructs. There was an average anionic *ζ*-potential of −19.3 ± 1.3 mV, and 87 ± 6.2 stochastically oriented ligands (Cet per TR-PIN = 24.5.0 ± 3.0, HT per TR-PIN = 30.9 ± 1.5, and TZ per TR-PIN = 34.1.0 ± 3.2), on the surface of nanoconstructs. For enhanced PDT efficacy, liposomal entrapped PS must be delivered and accumulated selectively in targeted tumor cells, to avoid toxic effects in normal tissues. The liposomal membrane provides numerous immobilization sites for recognition moieties such as antibodies, ligands, peptides, and electric charges [33], which over the past few decades have provided innovative solutions for improved binding of multiple payloads to cancer cells and circumventing off-target phototoxicity using photoactivable liposomal-based nanoconstructs for cancer cell targeting and the delivery of therapeutics [31–33].

Heterogeneous tumors such as PDAC exhibit patterns of tumor-associated cell surface receptors (EGFR, TfR, and HER-2) over-expression, and can be selectively targeted using PDT, directed against these receptors. Specific recognition of multiple cell surface targets may increase the specificity of drug delivery and treatment efficacy in heterogeneous tumor environments, thereby ultimately mitigating treatment escape. Using the established EGFR (1.7 × 10^5^ EGFR/cell) expression levels in MIA PaCa-2 cells we approximated that MIA PaCa-2 cells also express TfR (1.9 × 10^6^) and HER-2 (3.7 × 10^4^), which is consistent with our previous investigations [30, 32]. Similarly, relative cell surface expression levels of EGFR, HER-2, and TfR in PCAF cells are approximated using flow cytometry to be 4.8 × 10^4^, 1.5 × 10^6^, and 6.7 × 10^4^, respectively. It was found that the simultaneous targeting of three receptors demonstrates significantly higher cellular binding of TR-PINs, relative to the EGFR, TfR, and HER-2 hyperexpression in MIA PaCa-2 cells. Triple-receptor targeting resulted in 45-fold (MIA PaCa-2cells) improvements in binding with targeting when compared with the untargeted-PSNs (Figure 2(D)). CHO-WT cells, being null for the three receptors do not show enhancements in binding with targeting using TR-PINs. Similar results of higher binding with triple targeting employingTR-PINs were observed previously for a panel of cell lines including A431, T47D, SKOV-3, MIA PaCa-2, and SCC-9, in comparison to mono receptor targeting with the EGFR-specific PINs [30]. Furthermore, we compared the efficacy of molecularly targeted TR-PINs with an untargeted BPD-PC containing PSNs in MIA PaCa-2 cells (high in EGFR, TfR, and HEER-2) using 690 nm light at an irradiance of 150 mW/cm^2^ and a fluence of 20 J/cm^2^. Targeting improved the efficacy of photodestruction significantly compared to the untargeted PSNs (Figure 2(E)). We also compared the efficacy of TR-PINs with an untargeted BPD-PC containing PSNs in MIA PaCa-2 cells (high in EGFR, TfR, and HER-2) using 690 nm light at an irradiance of 150 mW/cm^2^ and a fluence of 20 J/cm^2^. Triple targeting using TR-PINs improved the efficacy of photodynamic activation compared to the untargeted PSNs (Figure 2(E)), which is consistent with its superior binding efficiencies (Figure 2(D)). For all TR-PINs concentrations tested, no dark toxicity was observed [30].

### 2.2 NIR light-mediated photodynamic treatment of 3D heterocellular Panc spheroids

**Figure 3:**
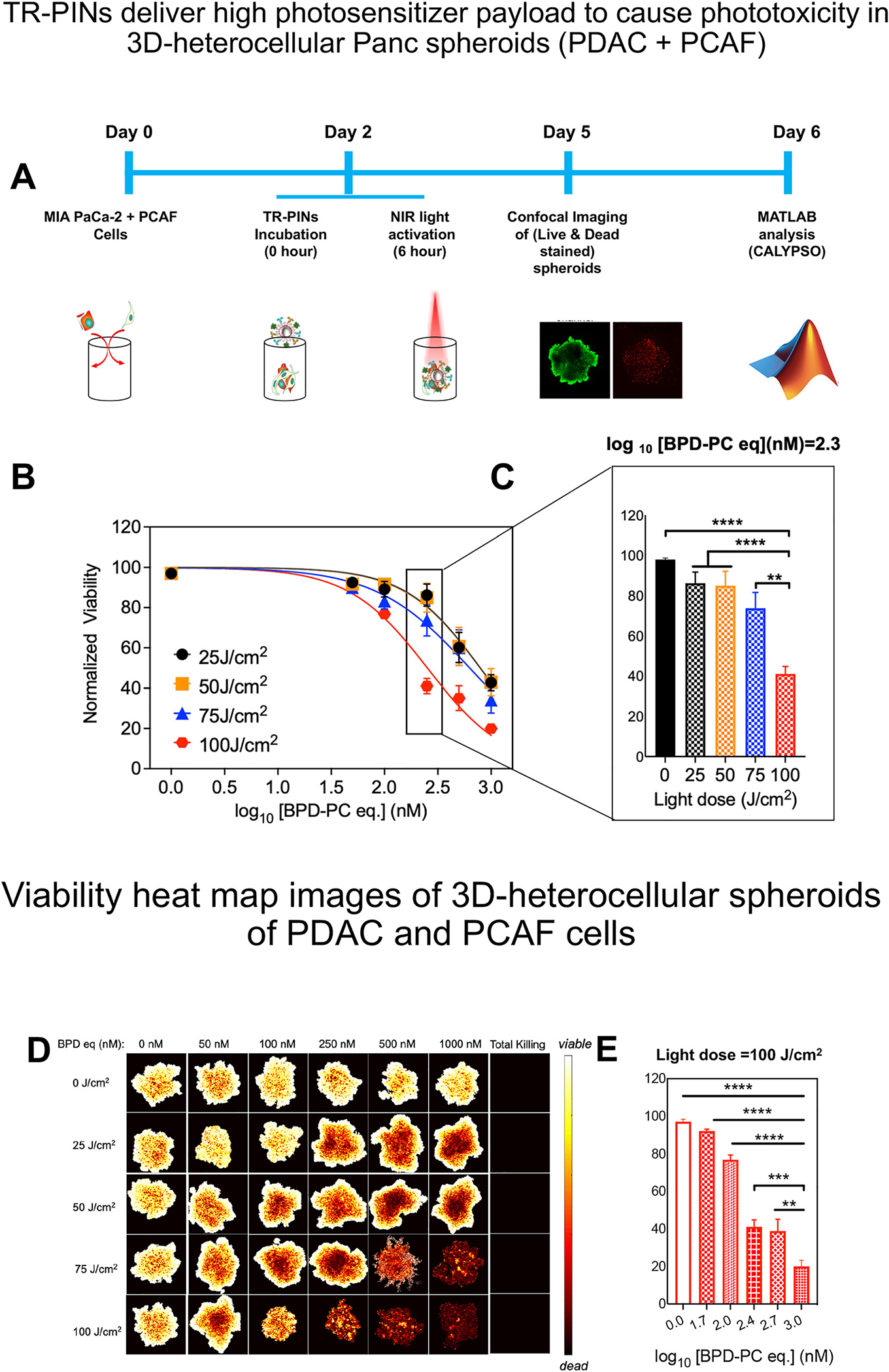
Schematic representation of the culturing and treatment of heterocellular Panc (PDAC-PCAF) spheroids followed by an imaging-based analysis of treatment response. A comprehensive image analysis procedure for structurally complex organotypic cultures was used for the quantitation of fractional viability of spheroids following NIR photodynamic activation (A) using TR-PINs and PSNs (B). The NIR photodynamic activation regimen used was 690 nm light irradiation with 25 or 50 or 75 or 100 J/cm^2^ at 150 mW/cm^2^. Quantitation of fractional viability of MIA PaCa-2 and PCAFs spheroids following NIR photodynamic activation (25, 50, 75, 100 J/cm^2^) at a log_10_ [BPD-PC] (nM) = 2.3 (250 nM of BPD-PC equivalent) using TR-PINs (C). Viability heatmap images of heterocellular (PDAC + PCAF) spheroids following NIR photodynamic activation of TR-PINs with increasing concentrations of the photosensitizer BPD-PC (D). Quantitation of fractional viability of spheroids following NIR photodynamic activation (100 J/cm^2^) using TR-PINs (E). (mean ± SEM; *n* = 9–12 for b–e; one-way ANOVA with a Tukey post-test; ****P ≤ 0.0001, ***P ≤ 0.001, **P ≤ 0.01).

NIR light-triggered PIT combines the advantages of the targeting and NIR light, conferring the specificity with the cytotoxicity of PDT to impart rapid and highly selective cell death. However, targeted destruction with the higher specificity becomes the central challenge, while addressing the resistance that arises from receptor heterogeneity. Multiple studies have reported positivity up to 95% for EGFR [34, 35] and 69% for HER-2 [36, 37] among patients with pancreatic tumors. The expression makes EGFR and HER-2 potential targets for light activatable molecular therapies. Because TfR over-expression has also been reported in PDAC, we have included TfR as an additional target. We had shown in earlier reports that TR-PINs (EGFR, HER-2, and TfR specific) exhibit expanded cancer cell binding specificities, enhanced cellular uptake, and superior PDT response compared to the single receptor-targeted therapy (specific for EGFR only) when studied in complex heterogeneous tumor models comprising MIA PaCa-2 cells and low-EGFR-expressing T47D or SKOV-3 cells [30]. Considering that EGFR, HER-2, and TfR overexpression is prevalent not only in PDAC but also in PCAFs cells, we thus further evaluated the specificity of TR-PINs for PS delivery and PDT efficacy in a more complex heterocellular tumor model of PDAC and PCAFs. Heterocellular spheroids of human PDAC (MIA PaCa-2 cells) and human PCAF cells are referred to as Panc spheroids from hereon. Established heterocellular Panc spheroids (MIA PaCa-2 and PCAFs) were incubated for 6 h with untargeted-PSNs or TR-PINs (0–1000 nM equivalent of BPD-PC), washed three times to remove any unbound TR-PINs and then irradiated with varying light doses (25 or 50 or 75 or 100 J/cm^2^) at an irradiance of 150 mW/cm^2^. These parameters of incubation time and irradiance previously allowed us to achieve sufficient intracellular PS (BPD-PC) accumulation to enable a potential PDT-enhancement effect while remaining nontoxic for spheroids. Following PDT (Figure 3(A)), the spheroids were co-stained with propidium iodide (Dead) Calcein AM (Live) reagents before single-plane confocal imaging. Quantitative fractional viability heatmap images were generated using a comprehensive high-throughput image analysis procedure or structurally complex organotypic cultures for the viability assessment of the tumor spheroids (Figure 3(B)) [38].

In the absence of photoactivation, neither untargeted PSNs nor TR-PINs exerted any significant toxic effects on heterogeneous Panc spheroids (Supplementary Figure 1) [30, 32]. Untargeted PSNs also did not show any significant phototoxicity even at the highest concentration of 1000 nM of BPD-PC equivalent (Supplementary Figure 2) at the highest light dose of 100 J/cm^2^ (150 mW/cm^2^). Irradiation (in the presence of TR-PINs) induced a PS dose-dependent increase in spheroid necrosis, which was significantly higher in spheroids treated with the light dose of 100 J/cm^2^ (Figure 3(B)). In the Panc spheroids of MIA PaCa-2 and PCAF, the EGFR-TfR-HER-2 specific TR-PINs were significantly more effective in killing cancer cells than the untargeted PSNs. Compared with low-TR-PINs concentration (50–100 nM of BPD-PC equivalent), NIRphotodynamic cytotoxicity using high-TR-PINs concentration (500–1000 nM of BPD-PC equivalent) was much stronger, exhibiting dose-dependence at all light doses used. The Panc spheroids viability only decreased to 23% after PDT with the TR-PINs concentration (100 nM of BPD-PC equivalent) at a light dose of 100 J/cm^2^ (150 mW/cm^2^) (Figure 3(A)). It is also evident that lower light doses (25 or 50 J/cm^2^; 150 mW/cm^2^) were not sufficient to cause significant differences in tumor cell viability when treated withthe higher TR-PINsconcentration (1000 nM ofBPD-PC equivalent) in the heterocellular Panc spheroid model. Even though a large proportion (43%) of tumor cells are eradicated from the Panc spheroids of MIA PaCa-2 and PCAFs, there are still residual cells remaining. Factors including differences in light distribution and the reduction in the rate of ^1^O_2_ production [30] (in the presence of a high number of ligands as in the case of TR-PINs) may influence the improved PDT in hetero cellular 3D spheroids. In the absence of direct PS-only control it is difficult to make a definitive statement, as 3D tumor models, recapitulating the *in vivo* TME to a great extent are heterogenous. Like a “real” tumor, the distribution of PS and light are not identical from cell to cell. All these results in heterogeneous outcomes. Understanding the mechanisms for why there remain residual tumor cells following treatment in the heterocellular spheroids is critical and serves as the focus of future studies.

Employing a light dose of 100J/cm^2^ (150mW/cm^2^) with higher TR-PINs concentrations (1000 nM of BPD-PC equivalent), led to an 80% reduction in Panc spheroid viability after PDT (Figure 3(C)), suggesting potent cytotoxic effects of using a light dose of 100 J/cm^2^. Moreover, ~60% reduction in spheroid viability was also observed after PDT with the TR-PINs (250 nM of BPD-PC equivalent) at a similar light dose of 100J/cm^2^ (Figure 2(D)), suggesting a combination of optimal TR-PINs concentration of 250 nM (BPD-PC equivalent) and a light dose of 100 J/cm^2^ may provide a better opportunity (Figure 3(D)) to understand *PDP* of antitumor immune responses. Thus, we selected TR-PINs (250 nM of BPD-PC equivalent) for subsequent experiments. Together, these findings provide compelling evidence for the potential of TR-PINs to enhance PDT efficacy through a light dose-enhancement effect, encouraging further *in vivo* investigations.

### 2.3 TR-PINs mediated induction of ICD

Apart from direct tumor cell death, PDT has been reported to induce ICD, characterized by the exposure or the release of DAMPs from dying cells at the site of tumor irradiation [5, 39,40]. These molecules alert the innate and adaptive arms of the immune system about the tumor by triggering local inflammation. DAMPs bind to cellular receptors (Toll-like receptors) and activate the innate immune cells such as macrophages or DCs which are highly specialized for presenting antigens to T cells, leading to T cell priming and enhanced ability to perform tumor cell killing. PDT-mediated ICD induction seems to be dependent on the type of PS (its cellular localization and PS concentration), light dose, DLI, and tumor model among other factors [3]. Therefore, in Panc spheroids we explored the ability of TR-PINs to induce ICD via the expression of previously reported DAMPs that are considered as “hallmarks of ICD,” including Hsp60, Hsp70, CRT, and HMGB1. We evaluated the expression kinetics of these molecules by applying varying light doses (25–100 J/cm^2^ at an irradiance of 150 mW/cm^2^) and analyzing the expression patterns at different time points (1–72 h post-PDT) using multi-color flow cytometry. Using gating strategies shown in Supplementary Figure 3(A), we detected surface expression of Hsp60, Hsp70, CRT, and intracellular expression of HMGB1.

**Figure 4:**
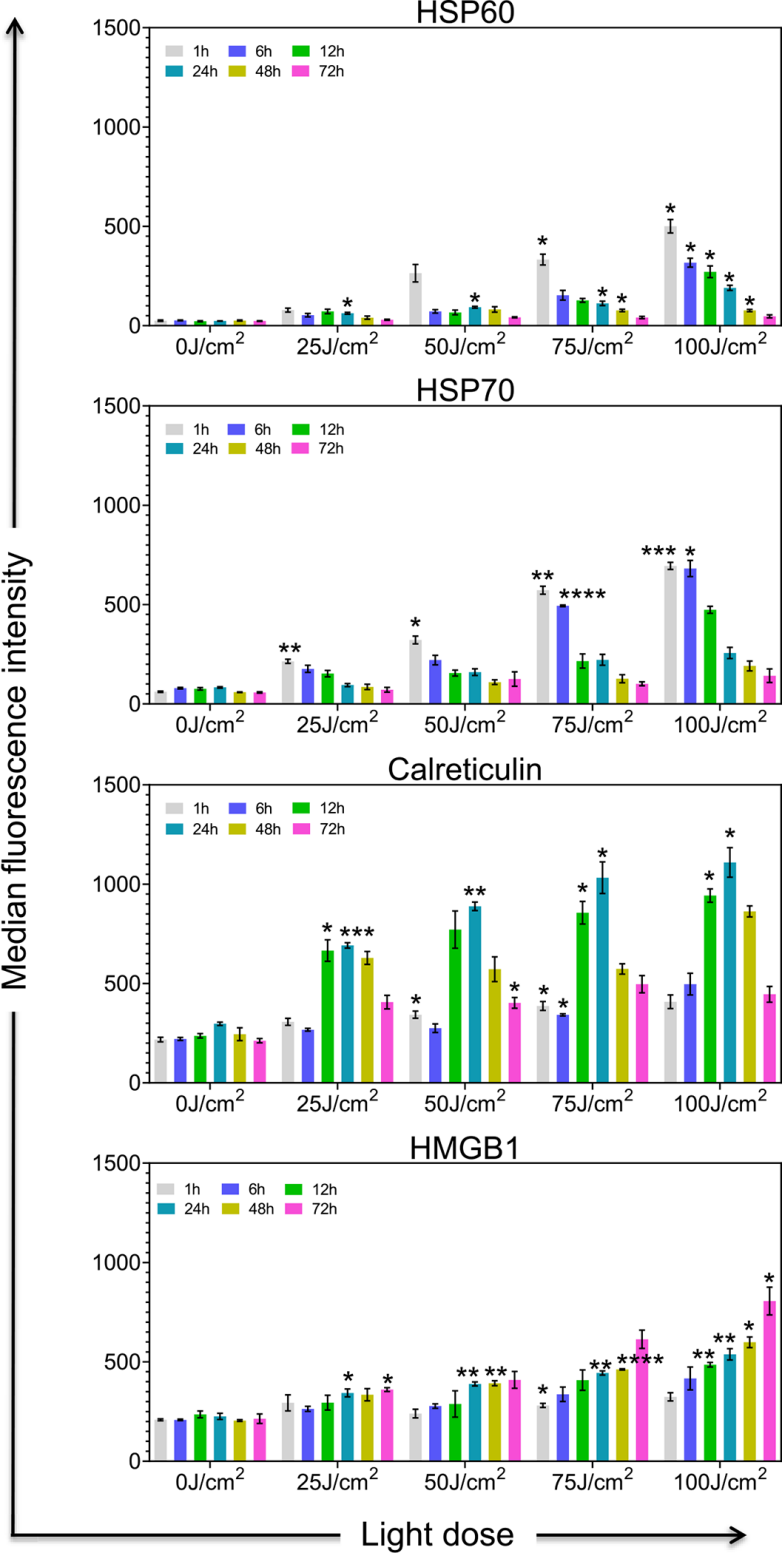
Expression of TR-PINs induced biological markers of immunogenic cell death in Panc spheroid cultures. NIR activation of TR-PINs induces cell surface exposure of Hsp60, Hsp70, Calreticulin, and the intracellular expression of HMGB1 in Panc (MIA PaCa-2 and PCAF) spheroid cultures in a light dose and time-dependent manner. Data are representative of three independent experiments done in duplicates. Expression levels of Hsp60, Hsp70, Calreticulin, and HMGB1 were determined by flow cytometry calculated as the median fluorescence intensity (MFI) after subtraction of the isotype controls MFI at 1, 6, 12, 24, 48, and 72 h after NIR activation of TR-PINs. Graphs with error bars indicate mean ± SEM from three independent experiments. Statistical significance was determined by a one-way ANOVA and Tukey’s posthoc test. Asterisks denote statistical significance (*P < 0.05, **P < 0.005, ***P < 0.0005, ****P < 0.00005). The NIR photodynamic activation regimen used was 690 nm light irradiation with 25 or 50 or 75 or 100 J/cm^2^ at 150 mW/cm^2^. Two hundred and fifty Newton-meters of TR-PINs (BPD-PC equivalent) were used.

Our data show that illumination of TR-PINs was able to induce the expression of Hsp60, Hsp70, CRT, and HMGB1 in a manner dependent upon light dose, PS concentration, and time, suggesting that TR-PINs mediated ICD (Figure 4 and Supplementary Figure 3(B) and (C)). Median fluorescence intensities (MFI) of CRT were comparatively higher than respective MFI of Hsp60, Hsp70, and HMGB1 for all light doses and TR-PINs concentrations. Hsp60 and Hsp70 were increased as early as 1 h post-PDT, with peak expression for both at 1–6 h for both and decreasing at 72 h relative to untreated controls or TR-PINs without light activation (data not shown) (Figure 4). Both the 75 and 100 J/cm^2^ light doses were also effective in inducing high levels of Hsp60 and Hsp70 at 1 and 6 h. The normal physiological role of Hsp60 and Hsp70 is to protect cells exposed to stressful conditions by safeguarding cell integrity and maintaining functional signaling pathways that are critical for cell survival and normal cell function [41, 42]. The protective response of Hsps after PDT seems to depend upon their cellular localization; intracellular localization appears related to antiapoptotic function, whereas extracellular Hsps or membrane-bound Hsps mediate immunological functions [43, 44]. Oxidative damage to cells by PDT-induced RMS modifies cellular proteins via fragmentation, cross-linking, unfolding, and aggregation; in this situation, Hsps identify unfolded proteins and help to either refold them or remove them via complex proteolytic systems. However, excessive accumulation of unfolded proteins in PDT treated cells can overwhelm the capacity of Hsp-mediated proteolytic pathways to repair or remove the abnormal proteins, leading to the formation of aggregates that are toxic to the cells. Previous studies have demonstrated PDT-mediated expression of Hsp60 [12, 45] or Hsp70 [12, 45–49] in various tumor cell line models *in vitro*. Consistent with our results, those studies also showed a temporal expression pattern of Hsps with more pronounced effects seen at highly cytotoxic PDT light doses [12, 46–49]. As mentioned before, early expression of membrane-bound Hsp60 and Hsp60 are powerful stimulants of antitumor immunity, helping to enhance TAAs and tumor cell killing by cytotoxic CD8^+^ T cells [43, 44].

In our study, CRT expression was significantly upregulated at 12–24 h compared to untreated controls or TR-PINs (Figure 4) without light activation, with peak expression at 24 h with increasing light dose. HMGB1 expression showed a slower but steady increasing trend from 1 to 72 h with high expression at 72 h in a light dose-dependent manner (Figure 4). CRT is usually located in the lumen of the endoplasmic reticulum, and it translocates to the cell surface during an immunogenic response. The cell surface expression of CRT sends “eat me” signals to phagocytic immune cells such as macrophages or DCs and helps these cells for the subsequent cross-presentation of tumor antigens to T cells. In order to be detected by phagocytic immune cells or other innate immune cells, dying cells must emit signals in addition to CRT. The release of HMGB1 from cancer cells undergoing ICD involves the permeabilization of both the nuclear and the plasma membranes that enables the translocation of the protein from the nucleus to the cytoplasm, followed by freeing into the extracellular space [50, 51]. Extracellular HMGB1 can bind multiple cell surface receptors to induce immune stimulation. PDT-mediated HMGB1 and CRT have been well described in previous studies [46, 52–56]. In our analysis, we were detecting intracellular HMGB1 levels which may not reflect its release from dying cells. These data also show that expression of Hsp60 and Hsp70 was more rapid whereas CRT or HMGB1 showed delayed expression during TR-PIN mediated ICD activation. The TR-PINs’ ability to induce Hsp60, Hsp70, CRT, and HMGB1 shows the potency of ICD in these pancreatic *in vitro* cultures and also highlights the possibility of immune stimulation.

### 2.4 TR-PINs mediated T cell activation and antitumor reactivity

**Figure 5:**
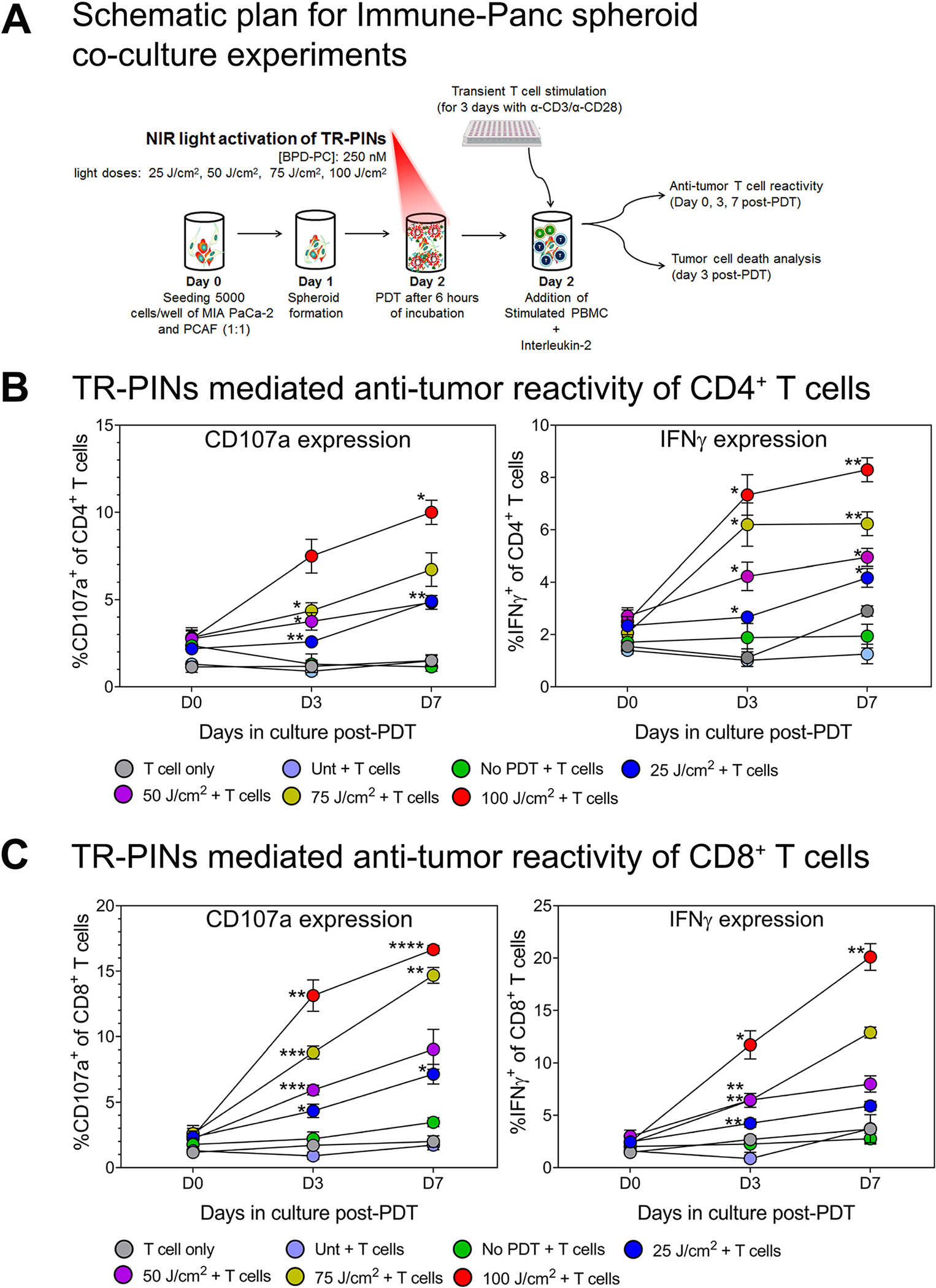
TR-PINs mediated priming of antitumor T cell reactivity in Immune-Panc spheroid co-cultures. (A) MIA PaCa-2 and PACFs were cultured and allowed to grow for 48 h before co-culture with peripheral blood mononuclear cells (PBMC).PBMC were seeded in 6-well plates with plate-bound anti-CD3 (overnight), anti-CD28, and IL-2, and T cells were allowed to proliferate for three days before addition to 3D spheroid cultures. This was done using a previous protocol with slight modifications [57, 58]. Once the spheroids were exposed to varying light doses, PBMC consisting mainly of mildly stimulated T cells were added to the cultures and allowed to remain for seven days. T cell priming was evaluated at day 3 and 7 post-PDT by analyzing the surface expression of degranulation marker CD107a and intracellular expression of INF*γ*. Also, in the same cultures, spheroid cell death was evaluated by flow cytometry analysis on day 3. The expression of CD107a and INF*γ* from day 0 in culture to day 7 was evaluated in (B) CD4^+^ T cells and (C) CD8^+^ T cells using multi-color flow cytometry. Data are means ± SEM from three to four independent experiments done in duplicate. Statistical significance was determined by a one-way ANOVA and Tukey’s posthoc test. Asterisks denote statistical significance (*P < 0.05, **P < 0.005, ***P < 0.0005). The NIR photodynamic activation regimen consisted of 690 nm light irradiation with 25 or 50 or 75 or 100 J/cm^2^ at 150 mW/cm^2^. TR-PINs (BPD-PC equivalent) were used at a concentration of 250 nM. All experimental conditions shown involved co-cultures Immune-Panc spheroids along with the addition of the BPD-containing TR-PINs, except for one condition with PBMC only (*T cell only*), and another with untreated Immune-Panc spheroids with added immune cells but no photosensitizer (*Unt + T cells*).

In order to study the NIR-TR-PINs activation of T cells, we used Immune-Panc spheroids (Panc spheroids combined with immune cells; MIA PaCa-2-PCAF and PBMC) (Figure 5(A)). This model was set up based on previously published protocols with slight modifications [57–60]. Allogenic PBMC were isolated from healthy human buffy coats and stimulated with anti-CD3 and anti-CD28 to mildly activateT cells for three days. In parallel, Panc spheroids (MIA PaCa2-PCAFs) were cultured (1:1) for two days until they grew to an optimum size. Then Panc spheroids were exposed to TR-PIN mediated photodynamic activation on day 2 and immediately, PBMC were added to the spheroid cultures (Immune-Panc spheroids) with an effector (T cell) to target (Panc spheroid cell) ratio of 5:1 and allowed to be in culture in the presence of IL-2 for seven days [57, 58]. The medium, including IL-2, was refreshed every three days. To demonstrate that this system supports T cells priming and expansion *in vitro* in the presence of Panc spheroids, we quantified interferon-gamma (INF*γ*) [58, 61, 62] and the degranulation of the cytolytic marker CD107a [58, 61–63] on CD4^+^ T cells and CD8^+^ T cells at baseline (day 0 of Immune-Panc spheroids in co-cultures), day 3 and day 7 using flow cytometry; our gating strategies are shown in the Supplementary Figure 4. We used TR-PINs at a concentration of 250 nM (the equivalent of BPD-PC) for all experiments mentioned in this section with varying light doses (25 or 50 or 75 or 100 J/cm^2^; 150 mW/cm^2^).

Light-induced activation of TR-PINs significantly increased the number of both INFγ and CD107a expressing CD4^+^ T cells (Figure 5(B)) and CD8^+^ T cells (Figure 5(C)) from day 0 to day 7 in co-cultures as compared to untreated controls or T cells alone. This increase of INFγ and CD107a positive T cells was more pronounced with increasing light dose and time in culture. IFNγ is produced by T cells in response to a variety of inflammatory or immune stimuli and has shown particular importance in tumor immune-surveillance [61]. Tumor cells can be recognized and killed by CD8^+^ effector T cells with help from CD4^+^ helper T cells, mainly through the immune secretion of lytic granules that kill target cells [64, 65]. This process involves the fusion of the granule membrane with the cytoplasmic membrane of the T cell, resulting in surface exposure of lysosomal-associated proteins that are typically present on the lipid bilayer surrounding lytic granules, such as CD107a [66]. Therefore, membrane expression of CD107a indicates cytotoxic degranulation and constitutes a marker of immune cell activation associated with antitumor immune reactivity. Our data show that INFγ and CD107a are upregulated, thereby suggesting that TR-PINs mediated T cell activation and enhanced effector antitumor reactivity. T cell activation and antitumor immune reactivity induced after PDT was reported in previous studies in preclinical models. Wachowska et al. showed that Photofrin-PDT leads to strong specific antitumor immune responses along with increased production of IFNγ and upregulation of CD107a in both CD4^+^ and CD8^+^ T lymphocytes of mice [63]. Another study that incorporated redaporfin-PDT in mice bearing CT26 tumors demonstrated an increased percentage of IFNγ-producing CD4^+^ and CD8^+^ T cell populations, highlighting the PDT mediated activation of antitumor T cells [67]. Our data add to previous findings by showing that priming and expansion of antitumor T cells are associated with the induction of ICD in pancreatic spheroid cultures that mimic a tumor immune microenvironment. The highest light dose that was successful in inducing potent ICD response (Figure 4) was also able to show enhanced T cell priming (Figure 5(B) and (C)).

### 2.5 Enhanced effects of PDT and T cell-mediated tumor cell killing

**Figure 6:**
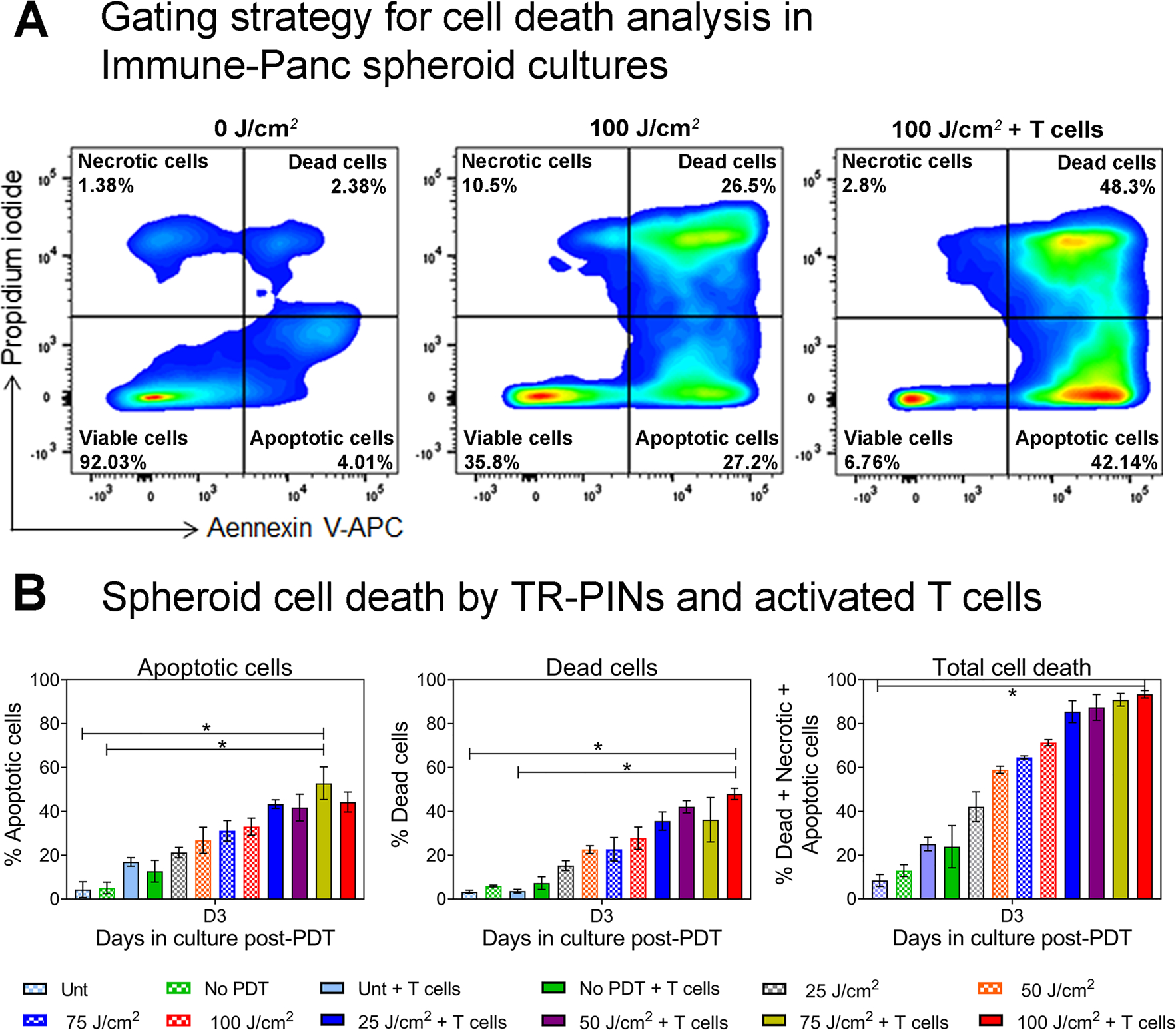
Synergistic effects on MIA PaCa-2 – PCAF cell killing by TR-PINs and antitumor reactive T cells. Analysis of cell death was performed using Annexin V and Propidium Iodide (PI) staining. (A) As shown in the gating strategy for cell death analysis, Panc spheroids were double-stained with Annexin V and PI and analyzed using flow cytometry. Four populations were identified as viable cells, apoptotic cells, dead cells, and necrotic cells as indicated in the flow cytometry plots. Quantification of apoptotic cells, dead cells, and total cell death (sum of apoptotic and dead cells including necrotic cells) under different culture conditions, is shown in figure (B). Data are means ± SEM from three independent experiments done in duplicates. Statistical significance was determined by a one-way ANOVA and Tukey’s posthoc test. Asterisks denote statistical significance (*P < 0.05). The NIR photodynamic activation regimen used 690 nm light irradiation with 25 or 50 or 75 or 100 J/cm^2^ at 150 mW/cm^2^. TR-PINs (BPD-PC equivalent) were used at a concentration of 250 nM. ***Key to conditions:*** *Unt:* untreated Panc spheroids (MIA PaCa-2 and PCAF) without T cells nor any nanoconstruct; *No PDT: Panc* spheroids with TR-PINs alone but no illumination; Unt + T cells: untreated Panc spheroids with T cells (Immune-Panc spheroids*); No PDT + T cells:* Immune-Panc spheroids with TR-PINS without illumination; 25, 50, 25, 75, or 100 J/cm^2^: Panc spheroids with TR-PINS and PDT at the indicated dose of light without T cells; 25J/cm^2^ + T cells, 50J/cm^2^ + T cells, 75J/cm^2^ + T cells, or 100 J/cm^2^ + T cells; Immune-Panc spheroids with TR-PINS and PDT at the indicated dose of light.

Although TR-PINs were able to exert efficient cell killing at the highest light doses in our initial experiments, we did not find complete tumor cell killing by TR-PINs after PDT (Figure 2(A)). Even at the highest dose of TR-PINs (1000 nM equivalent of BPD-PC) and light (100 J/cm^2^; 150 mW/cm^2^), there were about 20% of viable tumor cells in the Panc spheroids at day 3 post-PDT (Figure 2(A)). Moreover, ~60% reduction in spheroid viability was also observed after PDT with the TR-PINs (250 nM of BPD-PC equivalent) at a similar light dose of 100 J/cm^2^ (Figure 2(D)). Thus, we evaluated the enhanced effects of both PDP with TR-PINs (250 nM of BPD-PC equivalent) and T cells (CD8^+^ T cells) to exert efficient cellular cytotoxicity. As depicted in the schematic in Figure 5(A), tumor cell death in Immune-Panc spheroids was evaluated on day 3 after NIR activation of TR-PINs using flow cytometry. Different cell death profiles including necrotic, apoptotic, and dead cells were estimated with propidium iodide and annexin V staining as shown in Figure 6(A). The percentage of apoptotic cells was higher in Immune-Panc spheroids exposed to 75 J/cm^2^ compared to untreated controls (Figure 6(B)). However, the percentage of dead cells was higher in Immune-Panc spheroids cultures treated with 100 J/cm^2^, and the same cultures showed the highest percentage of complete cell death (taken as the sum of apoptotic, necrotic, and dead cells) by day 3 post-PDT. It is interesting to note that only a minor fraction (<10%) of spheroid cells were viable at day 3.

The remaining fraction of surviving Panc spheroid cells could be a concern. However, reports of achieving 100% tumor cell death are not universal in two-dimensional (2D) or 3D *in vitro*. There are many reasons for this observation and heterogeneity even in cell lines unless they have been carefully derived and maintained for monoclonality. Cell killing in 3D models is typically less [8, 30, 68, 69], compared to tumor-killing efficiency in 2D monolayer tumor models [70]. A possibility that some cells were still alive in our Immune-Panc spheroids may be attributed to the fact that the *in vitro* model used here is a “hard to kill” 3D model as PCAFs support tumor cell growth and possibly create dense desmoplastic 3D structures [71, 72]. PCAFs could elicit a strong immune suppressive effect on T cells that lead to their apoptosis [73–75]. Finally, these are 3D models, recapitulating the TME to a certain extent of the *in vivo* situation, thus the distribution of PS and light are not identical from cell to cell. All these factors bring heterogeneity adding resistance to the outcome. However, it is interesting to note that TR-PIN-mediated T cells activation achieving a significant level of tumor cell killing in our tumor spheroid model. Further, T cells added to the in Immune-Panc spheroid co-cultures are not autologous immune cells, which might limit T cell’s ability to recognize tumor cells in our model.

Although we used a heterotypic 3D *in vitro* model of PDAC, the tumor heterogeneity or antitumor immune effects of PDP are not completely recapitulated by this model. Therefore, PDP-mediated immune-stimulatory effects (local as well as systemic immune effects) could be better understood in an *in vivo* experimental model. Because PDP-mediated immune-stimulatory effects are not limited to the area where light is applied; the immune priming is that what extends well beyond the irradiated site [3]. Previous studies in immunocompetent mouse models show that PDP induces both the local antitumor responses as well as subsequent systemic immune effects that take place at distant tumor sites [4, 76]. These systemic immune effects may be dependent on PDP’s ability to expand an effector memory T cell pool [77, 78] supporting the notion that PDP’s ability to control the meta static disease as evident by the preclinical tumor models [4, 76, 79–81]. In addition to the immune effects of PDP, there could be other remote priming effects that could control tumor metastases shown by our previous works in immunodeficient mouse models of PDAC [20]. Huang et al. demonstrated that PDP can mitigate drug delivery barriers in the TME to safely enhance the therapeutic window of FDA-approved nanoliposomal irinotecan in a preclinical model of PDAC that also prevented tumor relapse [20]. PDP’s ability to augment efficient drug delivery has been attributed to enhanced vascular and stromal permeability as shown by Obaid et al. [32]. Also, a consequent reduction in metastatic burden, reported [20], possibly through the regulation of CXCL12/CXCR7/CXCR4 axis [20, 22] which helps to normalize PCAFs preventing their involvement in tumor metastases.

## 3 Conclusions

The enhancement of the DAMPs and T cells consequent to PDP and how they impact the overall killing are examined in this study using a complex heterogeneous 3D spheroid model of PDAC either with or without immune cells. A nanotechnology-enabled strategy, providing evidence that TR-PINs intensifies PDT efficacy through the light dose-enhancement effect in heterotypic Panc spheroids of PDAC and PCAFs. PDP allowed not only effective uptake of the targeted nanoconstructs but also the priming process further to induced potent ICD highlighted by upregulation of Hsp60, Hsp70, CRT, and HMGB1. ICD mediated enhanced immunogenicity was able to efficiently prime CD4^+^ T cells and CD8^+^ T cells evidenced by the upregulation of INFγ and degranulation marker CD107a. These activated T cells recognized tumor cells and provided further killing of remaining MIA PaCa-2 and PCAF cells in Immune-Panc spheroids. While priming effects for immune enhancement with PDT have been reported previously, to our knowledge, the effect of multiple targeted PDP has not been reported. The triple receptor targeting, in addition to our findings of immune stimulation also addresses tumor heterogeneity. Although encouraging, much work is warranted in the future to validate this approach, including testing in appropriate *in vivo* models and quantifying the changes in PS delivery and immune cell activation along with long-term effects on survival.

## 4 Experimental section

Complete experimental details can be found in the Supplementary Material.

## Supplementary Material

revised sup mat
