# Supplementary material for: Photodynamic priming with triple-receptor targeted nanoconjugates that trigger T cell-mediated immune responses in a 3D in vitro heterocellular model of pancreatic cancer
Source: Nanophotonics. Author manuscript; Available in PMC 2023 Jul 21. (PMC10361703; doi:10.1515/nanoph-2021-0304)
Supplement: revised sup mat [file NIHMS1913185-supplement-revised_sup_mat.pdf]

Supplementary Material

Pushpamali De Silva[a,†], Shazia Bano[a,†], Brian W. Pogue[b], Kenneth K. Wang[c] Edward V. Maytin[d], and Tayyaba Hasan[a,e*]

Photodynamic priming with nanoconjugates to trigger T cell-mediated immune responses in a 3D *in vitro* heterocellular model of pancreatic cancer

[a]*Wellman Center for Photomedicine, Massachusetts General Hospital and Harvard Medical School, Boston, MA 02114, USA*
[b]*Thayer School of Engineering, Dartmouth College, Hanover, New Hampshire 03755, USA*
[c]*Division of Gastroenterology and Hepatology, Mayo Clinic, Rochester, MN 55905, USA.*
[d]*Departments of Dermatology and Biomedical Engineering, Cleveland Clinic, Cleveland, OH*
[e]*Division of Health Sciences and Technology, Harvard University and Massachusetts Institute of Technology, Cambridge, MA 02139, USA*

**Corresponding Author: Tayyaba Hasan, PhD, Massachusetts General Hospital, 40 Blossom Street, BAR 314A, Boston, MA 02114. Phone: 617-726-6996; Fax: 617-Q4 724-1345; E-mail: thasan@mgh.harvard.edu*

† contributed equally to the work.

Article subtitle

Photodynamic activation of Verteporfin to boost anti-tumor immunity

Material and methods

All lipids, 1-arachidoyl-2-hydroxy-sn-glycero-3-phosphocholine (20:0 lyso PC), 1,2-distearoyl-sn-glycero-3-phosphocholine (18:0 PC (DSPC)), 1,2-dioleoyl-sn-glycero-3-phospho-(1'-rac-glycerol) (sodium salt)(DOPG), cholesterol, 1,2-distearoyl-sn-glycero-3-phosphoethanolamine-N-[methoxy(polyethyleneglycol)-2000 (DSPE-mPEG-2000) and 1,2-distearoyl-sn-glycero-3-phosphoethanolamine-N-[dibenzocyclooctyl(polyethylene glycol)-2000] (ammonium salt) (DSPE-PEG2000-DBCO) were obtained from Avanti Polar Lipids, and Verteporfin (Benzoporphyrin; BPD) was purchased from US Pharmacopeia.

Modification of proteins with NHS-PEG$_4$-N$_3$ and AF488-NHS

Trastuzumab (TZ, 145531.5 g/mol, Herceptin®; Genentech, FASTA sequence analysis) and human holo-transferrin (HT, 79680 g/mol; Sigma-Aldrich) were modified following our established protocol [1-3], through the conjugation of N-hydroxysuccinimidyl azido poly-ethylene glycol (NHS-PEG$_4$-N$_3$, 88.37 g/mol; Thermo Scientific) to the lysine residues of the proteins. Briefly, NHS-PEG$_4$-N$_3$ (5-fold molar excess) was reacted to the protein solution (2 mg/ml in 1x DPBS) in the presence of NHS-ester of Alexa Fluor® 488 (AF-NHS; 643.4 g/mol). After 24 hours of orbital rotation, AF and PEG$_4$-N$_3$ conjugated proteins were purified to remove any free reactants. The molar concentrations (M) of the purified proteins including Cet (ε280 nm = 217,315 $M^{-1}.cm^{-1}$), HT (ε280 nm = 83,360 $M^{-1}.cm^{-1}$) or TZ (ε280 nm = 225,005 $M^{-1}.cm^{-1}$) was determined using Nanodrop One (Thermo Scientific) and stored at 4°C in dark.

Preparation of triple-receptor targeted photoimmuno-nanoconjugates (TR-PINs)

Prior to liposomal preparation, the carboxylate group of the photosensitizer benzoporphyrin derivative (BPD) was coupled to the hydroxyl moiety of 1-arachidoyl-2-hydroxy-sn-glycero-3-phosphocholine (20:0 lyso PC; 1249.72 g/mol), through Steglich esterification, following our established method [1, 2] to form lipid conjugated BPD (BPD-PC). For the preparation of photosensitizing nanoconstructs (PSNs), all the lipids including DSPC (790.14 g/mol), DOPG (797.02 g/mol), DSPE-mPEG-2000 (2803.79 g/mol), DSPE-PEG2000-DBCO (3077.80 g/mol) and Cholesterol (386.65 g/mol), were mixed with lipidated BPD (BPD-PC) and dried to form a thin film. The dried lipid films were hydrated with 1 ml of 1XDPBS and were subjected to freeze-thaw cycles. Multilamellar vesicles were then sequentially extruded through polycarbonate membranes (100nm, Avanti® Polar Lipids, Inc.) to prepare small unilamellar liposomes. Triple targeted photoimmuno-nanoconjugates (TR-PINs) were prepared by reacting appropriate volumes of Cet-AF-PEG$_4$-N$_3$, HT-AF-PEG$_4$-N$_3$, and TZ-AF-PEG$_4$-N$_3$ to the PSNs. The mixture was kept on rotation at room temperature and purified after 24 hours using size exclusion columns packed with Sepharose CL-4B (Sigma-Aldrich) pre-equilibrated with 1X DPBS. Purified fractions were stored in the dark at 4°C.

Physical characterizations

BPD-PC concentration (nM) within the liposomal nanoconstructs was determined by diluting PSNs or TR-PINs in DMSO and measuring the absorption spectrum using UV-Visible absorption spectrophotometry (ε687nm = 34,895 $M^{-1}.cm^{-1}$). The ligand (Cet, HT, TZ) density to the surface of PSNs was derived as described previously. TR-PINs were characterized with regards to their hydrodynamic diameter (nm), polydispersity index (PDI), and ς-potential (mV) using the Zetasizer Nano ZS Dynamic Light Scattering Instrument (Malvern Instruments, Ltd., Houston, TX). Measurements were performed in triplicates and values were reported as mean and standard deviation.

Cell Culture

MIA PaCa-2 cells (ATCC) and Pancreatic Cancer-Associated Fibroblasts (PCAF) were cultured in Dulbecco's Modified Eagle's medium (DMEM). The PCAF cells were a kind gift from Dr. Diane Simeone. Cryopreserved PBMC vials were thawed, and cells were prewarmed in RPMI 1640 (GIBCO) media. All media was supplemented with 10% heat inactivated Fetal Bovine Serum (FBS, Gibco, ThermoFisher) and 100 U/mL penicillin, 100 µg/mL streptomycin (Mediatech) in Corning T75 cell culture flasks (Corning), and maintained in a humidified CO_2 atmosphere at 37 °C. All cells were confirmed negative for mycoplasma when tested using the MycoAlert Plus mycoplasma kit (Lonza) and this was done routinely.

Preparation and photodynamic treatment of Panc spheroids

Suspended 3D Panc spheroids (MIA PaCa-2 and PCAF cells) were cultured in 96-well, round bottom ultralow attachment plates (Corning® Costar®) at 37°C. MIA PaCa-2 and PCAF cells were seeded at a density of 5000 cells per well (1:1 ratio) for 48 hours to self-assemble into single 3D spheroids. We labelled these spheroids, as Panc spheroids. Spheroids were then incubated with PSNs or TR-PINs at varying concentrations of BPD-PC (50 nM to

1000 nM). After 6 hours of incubation, spheroids were washed three times with serum-containing cellular media and irradiated using 690 nm laser light (Intense, North Brunswick) at an irradiance of 150 mW/cm^2 with varying light doses (25 J/cm^2 to 100 J/cm^2). Following photodynamic activation, cells were co-stained with LIVE (Calcein AM) & DEAD (propidium iodide) reagents at standard culture conditions according to the manufacturer's protocol to analyze the viability of treated cells. Fluorescence signals were recorded using an Olympus FV-1000 confocal microscope through a 0.16NA 4x air objective at λexc = 488 nm/λem = 520 nm (calcein) and λex = 559 nm/λem = 630 nm (PI). Brightfield images were acquired under 559 nm light. The acquisition was standardized for each nodule. All experimental conditions were performed with an n of 8-12 nodules. Comprehensive high-throughput image analysis (CALYPSO)[4] was used to generate heat map images and for quantifying the fractional viability

PBMC isolation
Healthy human buffy coats were purchased from BioIVT. The peripheral blood mononuclear cells (PBMC) fraction was isolated from buffy coats by Ficoll-Paque density gradient separation, which was then cryopreserved until later use.

Immune-Panc spheroid co-cultures
MIA PaCa-2 and PACFs were cultured as described above "Preparation of Panc spheroids". The spheroids were allowed to grow for 48 hours before co-culturing them with PBMC to make Immune-Panc spheroids. PBMC (2-3*10^6 cells/mL) were seeded onto 6-well plates (Corning) coated overnight with anti-CD3 (2 µg/mL of clone OKT3, eBioscience), and with anti-CD28 co-stimulation (2 µg/mL of clone CD28.2, eBioscience) in which T cells were allowed to proliferate for 3 days before addition to the Panc spheroid cultures. This was done using a previous protocol with slight modifications [5, 6]. Thus, after 3 days of mild T cell stimulation, PBMC were added to the PDT-treated Panc spheroids in an effector-to-target ratio of 5:1 [5, 6]. Interleukin (IL)-2 (150 U/mL) was added to the cultures to provide support for the T cells. The medium, including IL-2, was refreshed every 3 days and maintained in culture for 7 days.

Flow cytometric analysis
Binding of TR-PINs to PDAC and PCAF cells
For cellular binding of TR-PINs, single-cell suspensions of 50,000 cells/microcentrifuge tubes were incubated with the untargeted-PSN or TR-PINs (250 nM BPD-PC equivalent) in serum-containing culture media at 37°C for 30 min in the dark. For the approximation of expression levels of EGFR, TfR, and HER-2, MIA PaCa-2 and PCAF cells were incubated with 10 ug/ml of AF conjugated Cet, TF, or TZ at 37°C for 30 min in the dark. Following incubation, the cells were washed and resuspended in pre-cooled 1x DPBS. The fluorescence intensity of cell-associated BPD-PC and Alexa Fluor 488 was measured using the BD FACSAriaTM II flow cytometer (BD Biosciences®). Ten thousand events were recorded and gated for each group using a 405 nm laser and a 610 nm dichroic long-pass filter for BPD and a 450/40 nm filter for AL488. Median BPD-PC emission was quantified using FlowJo® software (version 10, BD). Data is presented as mean ± SEM from six biological replicates for each group.

Hsp60, Hsp70, Calreticulin and HMGB1 detection: Suspensions of MIA PaCa-2 – PCAF cells were incubated with the manufacturers' suggested dilution of fluorescently labeled primary monoclonal antibodies including anti-Calreticulin-PE (D3E6, Cell Signaling Technology), anti-HMGB1-PE (3E8, Biolegend), anti-HSP60-FITC (LK1, StressMarq) and anti-Hsp70-APC (REA349, Miltenyi Biotech) with relevant isotype controls. Intracellular labeling of HMGB1 was accomplished using fixed and permeabilized cells (pre-labeled for membrane markers) with the BD Cytofix/Cytoperm™ Fixation/Permeabilization Solution Kit (BD 554714) following manufacturer's protocol. Tumor cell suspensions were prepared from spheroids by enzymatic dissociation (Trypsin, Corning) into single cells**.**

Detection of cell death and apoptosis in Immune-Panc spheroid co-cultures: Suspensions of Immune-Panc spheroids were incubated with the manufacturers' suggested dilution of fluorescently labeled primary monoclonal antibodies including anti-CD45-PE-Vio770 (Miltenyi Biotech), anti- CD3-Vioblue (Miltenyi Biotech), anti-CD44-FITC (eBioscience), anti-326 (EpCAM)-APC-Vio770 (Biolegend) prior to staining with Propidium Iodide (Biolegend) and Annexin V-APC (Biolegend). This combination allows the separation of immune cells from MIA PaCa-2 – PCAF cells. Propidium Iodide and Annexin V-APC staining was done following manufacturer's protocol (Biolegend).

CD107a and Interferon gamma (IFNγ) staining: On day 0, 3 or 7, anti-human CD107a-PE (BD Bioscience) antibodies were added to the co-cultures with immune cells. After 1 hour of incubation, Golgi-Plug (1:1000, BD Bioscience) and Golgi-Stop (1:1500, BD Bioscience) were added to the co-culture and incubated for another 4 hours. Cells were washed twice in FACS buffer and incubated with the manufacturers' suggested dilution of fluorescently labeled primary monoclonal antibodies for surface markers including anti-CD45-Viogreen (Miltenyi Biotech), anti-CD3-Vioblue (Miltenyi Biotech), anti-CD4-PE-Vio770 (Miltenyi Biotech), anti-CD8-FITC (Miltenyi Biotech), and eBioscience™ Fixable Viability Dye eFluor™ 780 to exclude dead cells. Intracellular labeling of IFNγ (anti-IFNγ-APC, R&D Systems) was accomplished using fixed and permeabilized cells (pre-labeled for membrane markers) with the BD Cytofix/Cytoperm™ Fixation/Permeabilization Solution Kit (BD 554714) following the manufacturer's protocol. All fluorescently labeled cells were acquired on a BD FACSAriaTM II flow cytometer (BD Biosciences®) and analyzed FlowJo® software (version 10, BD).

Statistical analyses

All results are reported as mean ± standard deviation of the mean (SD) unless otherwise specified. Statistical tests were carried out using GraphPad Prism Version 9 (GraphPad Software Inc., La Jolla, CA, USA). Specific tests are indicated in the figure captions. All reported P values are two-tailed unless otherwise specified. Analyses were performed using nonparametric tests (Kruskal–Wallis and one-way ANOVA with appropriate post hoc test, Dunn's test was used for multiple comparisons). All statements of significance in the results were based on a threshold of $p<0.05$. No exclusion criteria were used.

References

1. Obaid, G., et al., Nanolipid Formulations of Benzoporphyrin Derivative: Exploring the Dependence of Nanoconstruct Photophysics and Photochemistry on Their Therapeutic Index in Ovarian Cancer Cells. Photochem Photobiol, 2019. **95**(1): p. 364-377.
2. Obaid, G., et al., Impacting Pancreatic Cancer Therapy in Heterotypic in Vitro Organoids and in Vivo Tumors with Specificity-Tuned, NIR-Activable Photoimmunonanoconjugates: Towards Conquering Desmoplasia? Nano Lett, 2019.
3. Bano, S., et al., NIR Photodynamic Destruction of PDAC and HNSCC Nodules Using Triple-Receptor-Targeted Photoimmuno-Nanoconjugates: Targeting Heterogeneity in Cancer. Journal of clinical medicine, 2020. **9**(8): p. 2390.
4. Bulin, A.L., M. Broekgaarden, and T. Hasan, Comprehensive high-throughput image analysis for therapeutic efficacy of architecturally complex heterotypic organoids. Sci. Rep., 2017. **7**(1): p. 16645.
5. Jenkins, R.W., et al., Ex Vivo Profiling of PD-1 Blockade Using Organotypic Tumor Spheroids. Cancer Discov, 2018. **8**(2): p. 196-215.
6. Dijkstra, K.K., et al., Generation of Tumor-Reactive T Cells by Co-culture of Peripheral Blood Lymphocytes and Tumor Organoids. Cell, 2018. **174**(6): p. 1586-1598.e12.

Supplementary Figures and Legends

Absence of dark toxicity of untargeted PSNs and the TR-PINs in 3D-heterocellular spheroids of PDAC and PCAF

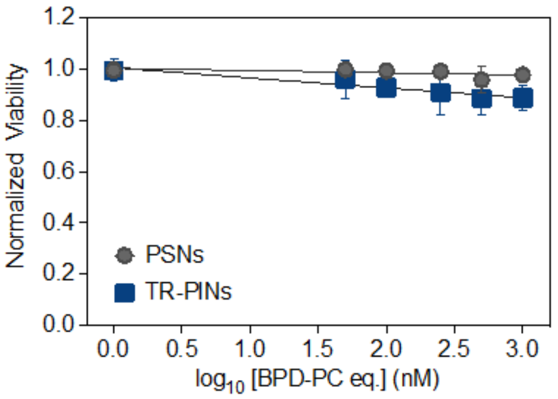

Supplementary Figure 1. Toxicity evaluation of Panc spheroids consisting MIA PaCa-2 and PCAFs after triple-receptor-targeted photoimmuno-nanoconjugates (TR-PINs) or photosensitizing nanoconstructs (PSNs) incubation in the absence of NIR-light. A comprehensive image analysis procedure for structurally complex organotypic cultures was used for the quantitation of fractional viability of spheroids, 72 hours following TR-PINs and PSNs incubation and washing. (Mean ± SEM; n = 9-12)

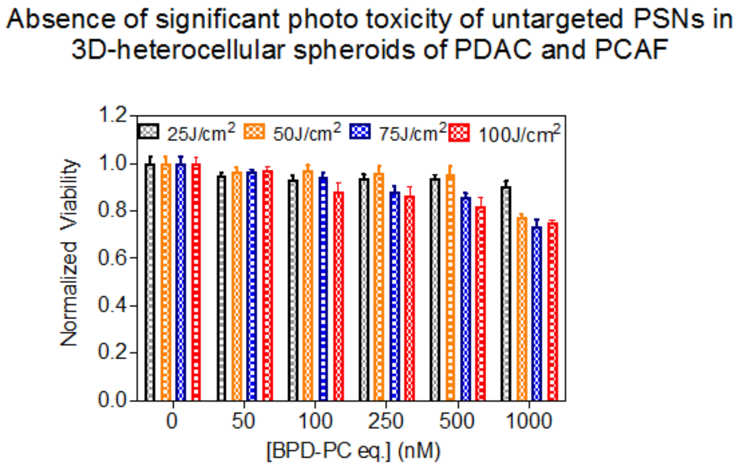

Supplementary Figure 2. Phototoxicity evaluation of Panc spheroids of MIA PaCa-2 and PCAFs after photosensitizing nanoconstructs (PSNs) incubation. A comprehensive image analysis procedure for structurally complex organotypic cultures was used for the quantitation of fractional viability of spheroids following NIR photodynamic activation, using PSNs. The NIR photodynamic activation regimen used was 690 nm light irradiation with 25 J/cm^2 or 50 J/cm^2 or 75 J/cm^2 or 100 J/cm^2 at 150 mW/cm^2. (Mean ± SEM; n = 9-12)

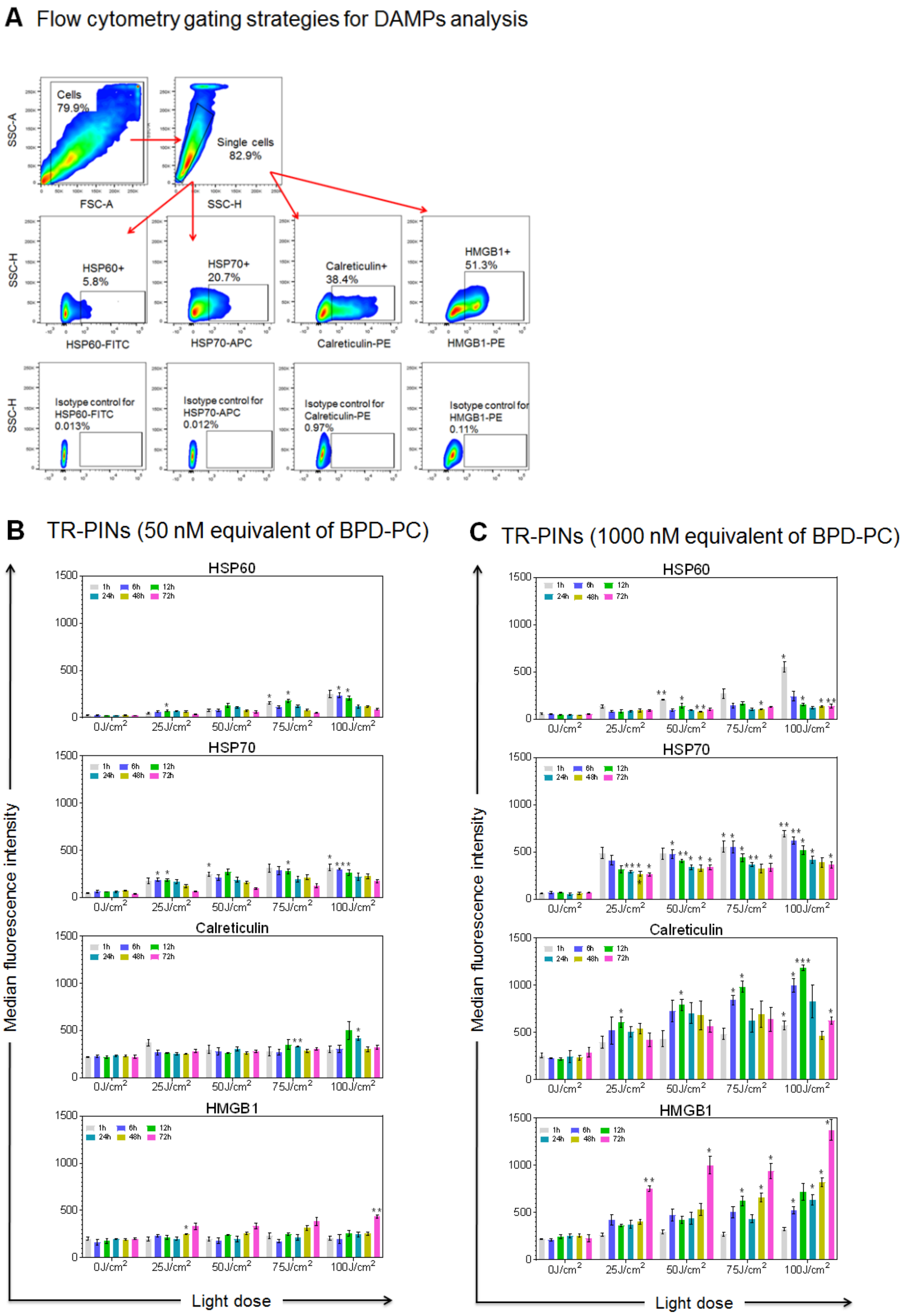

Supplementary Figure 3. Induction of TR-PINs mediated immunogenic cell death in Panc spheroids. NIR activation of TR-PINs induces cell surface exposure of Hsp60, Hsp70, Calreticulin and the intracellular expression of HMGB1 in Panc spheroids consisting MIA PaCa-2 and PCAFs in a light dose, TR-PINs concentration (equivalent to BPD-PC) and time dependent manner. **(A)** Representative flow cytometry plots depicting the gating strategies for the analysis of Hsp60, Hsp70, Calreticulin and HMGB1 comparing with the isotype controls for each marker. Data are representative of 3 independent experiments done in duplicates. Expression levels of Hsp60, Hsp70, Calreticulin and HMGB1 were determined by flow cytometry calculated as the median fluorescence intensity (MFI) after subtraction of the isotype controls MFI at 1 hour, 6 hour, 12 hour, 24 hour, 48 hour and 72 hour after NIR activation of TR-PINs. The NIR photodynamic activation regimen used was 690 nm light irradiation with 25 J/cm^2 or 50 J/cm^2 or 75 J/cm^2 or 100 J/cm^2 at 150 mW/cm^2. **(B)** [BPD-PC] (nM) = 2.3 (50 nM of BPD-PC equivalent) and **(C)** [BPD-PC] (nM) = 2.3 (1000 nM of BPD-PC equivalent) was used for the TR-PINs. The graphs with error bars indicate mean ± SEM from three independent experiments. Statistical significance was determined by a one-way ANOVA and Tukey's post-hoc test. Asterisks denote statistical significance (*P < 0.05, **P < 0.005, ***P < 0.0005).

Flow cytometry gating strategies for T cell activation

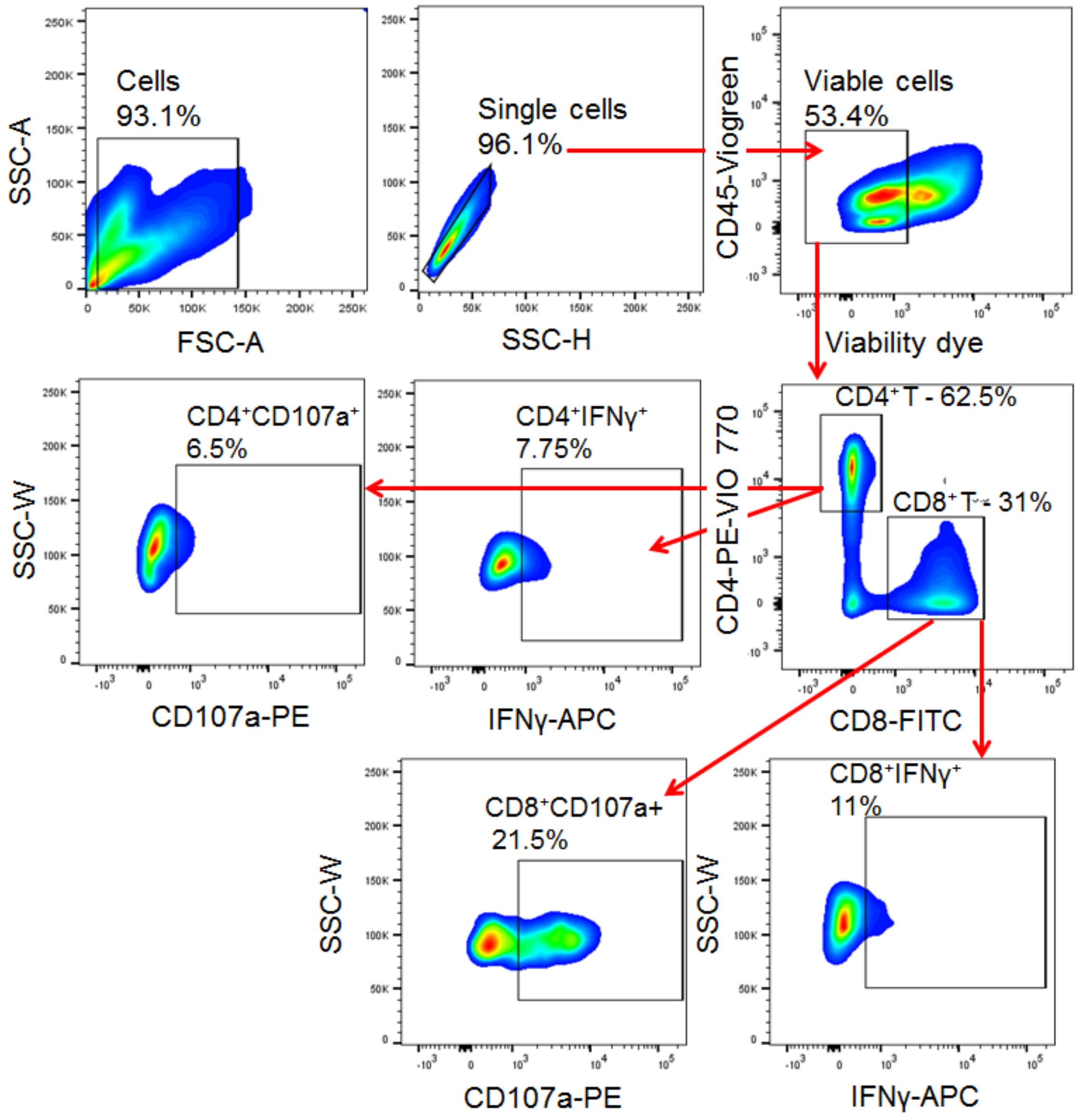

Supplementary Figure 4. Flow cytometry gating strategies for CD107a and INFγ expression on T cells in Immune-Panc spheroid co-cultures after NIR activation of TR-PINs. % of CD107a and IFNγ positive $CD4^+$ T cell and $CD8^+$ T cell fraction was quantified comparing with isotype controls for each marker on day 0, day 3 and day 7.
